# The construct validity of the Child Health Utility 9D-DK instrument

**DOI:** 10.1186/s12955-019-1256-0

**Published:** 2019-12-23

**Authors:** Karin Dam Petersen, Julie Ratcliffe, Gang Chen, Dorthe Serles, Christine Stampe Frøsig, Anne Vingaard Olesen

**Affiliations:** 1 Department of Business and Management, Faculty of Social Sciences, Aalborg University, Fibigerstræde 11, 9220 Aalborg East, Denmark; 2 Institute of Health Economics, Ternevej 31, 8240 Risskov, Denmark; 3 Health and Social Care Economics Group, College of Nursing and Health Sciences, Flinders University, Sturt North Wing (N206) GPO Box 2100, Adelaide, South Australia 5001 Australia; 4 Centre for Health Economics, Monash Business School, Monash University, 900 Dandenong Road, Caulfield East, VIC 3145 Australia; 5 Vocational Colleges, Østre Boulevard 10, 9600 Aars, Denmark; 6 Department of Civil Engineering, Faculty of Engineering and Science, Division of Transportation Engineering, Traffic Research Group, Aalborg University, Thomas Manns Vej 23, 9220 Aalborg East, Denmark

**Keywords:** Young adults, Adolescents, Health related quality of life, Outcome assessment, Patient-reported outcomes, CHU9D, PedsQL, High school, Construct validity

## Abstract

**Background:**

Relative to their application with adults there is currently little information about the application of preference-based health-related quality of life (HRQL) instruments among populations of young people. The Child Health Utility 9D (CHU9D) is a paediatric-specific generic preference-based HRQL instrument, recently translated and linguistically validated into Danish (CHU9D-DK). The purpose of this study was to investigate the construct validity of the CHU9D-DK in a sample of Danish high school students.

**Methods:**

All students attending a Danish High School were invited to participate in a web-based survey in January 2018 (*N* = 272). The survey included the CHU9D-DK, the young adult version of the Pediatric Quality of Life Inventory™ 4.0 Generic Core Scales (PedsQL), self-reported health status, presence/absence of disability/chronic diseases, life satisfaction, and socio-economic questions. CHU9D-DK utility scores were generated by employing the two scoring algorithms developed from adults in the UK and adolescents in Australia, respectively. Internal consistency, reliability and construct validity of the CHU9D-DK instrument were investigated.

**Results:**

Two hundred and twenty-eight (84%) students consented to participate and completed the survey. The mean ± (standard deviation) values of the CHU9D-DK utilities were 0.84 (0.11) when the UK adult algorithm was applied and 0.70 (0.22), when the Australian adolescent algorithm was applied. The mean PedsQL score was 82.32 (13.14). The CHU9D-DK showed good internal consistency reliability (Cronbach’s alpha = 0.803). Higher levels of health status and life satisfaction were significantly associated with higher CHU9D-DK utility scores regardless of which scoring algorithm was applied (*p*-values < 0.001). Students living with a disability/chronic disease exhibited significantly lower utility scores relative to their healthy peers (*p*-values < 0.05). Higher socio-economic status (approximated by financial situation and frequency of family vacations) was also associated with higher utility scores (p-values < 0.005).

**Conclusion:**

The CHU9D-DK demonstrated good psychometric performance overall and shows potential as a valid and reliable instrument for assessing the HRQL of Danish young people.

**Trial registration:**

ClinicalTrials.gov identifier: NCT03391999, Registered 15 October 2017.

## Background

The adolescent phase is a transitional stage of physical and psychological development that occurs during the period from puberty to legal adulthood (age of majority), which in Denmark is 18 years. Whilst adolescence is usually associated with the teenage years, its physical, psychological or cultural expressions may begin earlier and end later. For example, puberty now typically begins during preadolescence, particularly in females. Physical growth (particularly in males), and cognitive development can extend into the early twenties. Thus biological age provides only a rough marker of adolescence and young adulthood and scholars have found it difficult to agree upon a precise definition of adolescence [1].

Adolescence and young adulthood is also a period of multiple transitions involving education, training, and first employment, as well as changes from one living circumstance to another [2, 3]. This phase of life is critical for the individual’s future lifestyle and behaviour and may play an important role in the development and persistence of lifestyle diseases [4, 5]. In general, in contrast to adult populations, there is sparse information available about younger age groups’ health-related quality of life (HRQL) in Denmark, in particular in relation to individuals’ subjective assessment of their own HRQL through the use of validated instruments.

HRQL instruments can be divided into non-preference-based and preference-based instruments [6]. The unique feature of preference-based instruments is their scoring algorithms, which are typically generated from large general population samples and are based on the relative weights or utilities attached to HRQL states defined by the instrument on a cardinal scale, where 0.0 represent dead and 1.0 represents full health [6, 7]. Preference-based HRQL instruments can be applied to generate quality-adjusted life-years (QALYs). QALYs combines length of life and quality of life into a single composite measure of outcome, which is preferable for health economic evaluation [8].

The Child Health Utility 9D (CHU9D) is a relatively new preference-based instrument for the measurement and valuation of HRQL in children and adolescents developed in the United Kingdom (UK) in 2009 [9]. Relative to other preference based HRQL instruments, the CHU9D has the advantage that it was developed specifically for application in pediatric populations and young people were involved in its original development. The CHU9D was developed from its inception with young people using qualitative research methods about what quality of life means to them. The identified dimensions of HRQL within the CHU9D instrument emanate from young people’s descriptions of what HRQL means to them and how they would define it [10, 11].

Since its original development the CHU9D has shown good psychometric performance in samples of young people in other countries beyond the UK including translation and validation into China (CHU9D-CHN) and validation in Australia [12–14]. CHU9D has recently been translated and linguistically validated into Danish (CHU9D-DK). The translation and linguistic validation were performed by the professional language service company ICON Language services (certificate number 2920-TX-0002), which is an ISO 17100-certified translation provider, specializing in the translation of documentation related to global clinical research and in the translation and linguistic validation of patient-reported outcomes including utility instruments [15].

In Denmark, there is currently a dearth of validated instruments available for assessing HRQL in child and adolescent populations. This is particularly the case for preference-based instruments suitable for application in health economic evaluation [16, 17]. The main purpose of this study was therefore to investigate the construct validity of the newly translated and validated CHU9D-DK instrument in a young community-based sample of adult/adolescent high school students.

## Methods

### Sample

The school in which this study was undertaken was a randomly selected Danish high school among the 27 geographically accessible high schools to the University of Aalborg (the employment location of the lead researcher) in the Northern part of Jutland. The study was undertaken during the academic school year 2017/2018. In January 2018 following agreement from the school principal to participate in the research, all 272 students in the high school were invited via email to participate in a web-based survey of approximately 15 min duration. The survey was developed specifically for this study via the Scandinavian tool SurveyXact, a software package for creating and conducting customised questionnaire-based surveys [18]. By clicking on a person-unique link provided in the email, students completed the survey online. According to Danish and international laws, the students were informed about the purpose of the study and that it was voluntarily to participate. The voluntary completion of the survey by the students constituted their implied consent to participate in the study.

### Survey

The online survey contained three components. The main component comprised the Danish versions of the two generic HRQL instruments (CHU9D-DK and PedsQL - introduced in more detail below). The other components included students’ self-assessed general self-reported health status, whether they had a disability or chronic disease, their life satisfaction and two items about their family’s socioeconomic situation. General health was reported on an ordinal scale (Excellent, Very good, Moderate, Not so good or Bad). The presence or absence of disability or chronic diseases was reported as Yes or No. The student’s overall life satisfaction was reported on an ordinal scale ranging from 1 to 10, Not satisfied (1–3), Medium satisfied (4–7), Very satisfied (8–10) or Don’t know, which was coded as missing.

Finally, family socio-economic status was approximated using two questions that have previously been applied in this context in the literature [19–21]. The first question was about financial situation. The students were asked, “Compared to other families where you live, do you think your family has (a lot of money, a reasonable amount of money, neither a lot or a little amount of money, little money or very little money)”. This item has been used in other Danish national investigations of children and adolescents welfare and well-being [19]. The item has shown that those who themselves experience having ‘little’ or ‘very little’ money, to a high degree are also those, who in more objective calculations of poverty, had relatively few financial resources.

The second question was about vacation/holiday experience in the last year. Students were asked, “Have you been on vacation in the last year? (Holiday stay with a minimum of four nights outside your usual place of residence – disregard for weekend trips) (Yes, several times, Yes, one time, No)”. This item is similar to an item used in the widely validated Family Affluence Scale (FAS), designed for self-report by adolescents aged 11–17 years [20]. This item has also been used in Danish contexts before, where it has been showed to exhibit strong positive associations with family financial situation [21]. Background information relating to the students’ age, gender, school programme and year of study was also collected for each consenting respondent by extracting this information from the high school register.

#### Child Health Utility 9D (CHU9D)

The CHU9D has been validated for self-completion by young people aged 7–17 years [16], and it has also been adapted for use and successfully applied in young adult populations aged 18–29 years [22, 23]. The CHU9D contains nine main dimensions (worried, sad, pain, tired, annoyed, schoolwork/homework, sleep, daily routine, and activities), each with five increasing levels of severity/impairment see Appendix. The individual responses to the CHU9D were converted to utilities by application of the existing UK adult general population scoring algorithm based on the standard gamble method [24]. For comparative purposes the responses were also converted to utilities using the recently developed Australian (AUS) adolescent-specific scoring algorithm based upon the best-worst scaling method [25, 26]. Application of the UK/AUS adult/adolescent scoring algorithms results in CHU9D utility scores ranging from 0.3261/− 0.1059 (reflecting the respective utilities attached to the most severe CHU9D health state: “PITS”) to 1.000 (reflecting full health for both scoring algorithms).

#### Pediatric quality of life inventory (PedsQL) 4.0 generic Core scales

The PedsQL is a generic, non-preference-based 23-item instrument assessing four main health dimensions: ‘Physical Functioning’ (8 items), ‘Emotional Functioning’ (5 items), ‘Social Functioning’ (5 items) and ‘School Functioning’ (5 items). The last three dimensions can also form a ‘Psychosocial health’ dimension, whilst the first dimension can also be called the ‘Physical health’ dimension. Respondents rate their answers on a 5-point Likert scale with one of the following preferences: 0 ‘never a problem’, 1 ‘almost never a problem’, 2 ‘sometimes a problem’, 3 ‘often a problem’, 4 ‘almost always a problem’. Items are then reverse-scored and linearly transformed into a total score ranging from 0 to 100 (where 0 = 100, l = 75, 2 = 50, 3 = 25, 4 = 0). Higher total scores represent better HRQL. The mean total score is a summation of all the items over the number of items answered, thereby accounting for missing data if present. This total scale score measures overall generic HRQL [27, 28]. The PedsQL is available and linguistically validated into Danish and it has been applied previously in several Danish contexts [29–31]. The PedsQL young adult’s version (age 18–25) was applied in this study as a key component to assess construct validity through relevant empirical comparisons with the CHU9D-DK [32].

### Statistical analysis

Statistical analyses were performed using Stata version 14.2 (StataCorp LP, College Stadion, Texas, USA). Continuous variables were described as mean ± standard deviation (SD). Categorical variables were described as frequencies. Chi-squared tests were used to test differences in categorical variables, whereas Students t-test/ANOVA was used for continuous variables whenever appropriate. The distribution of utility scores was tested for normality using the Shapiro-Francia test. When the normality assumption was not met, a non-parametric test was used. A 5% significance level was used in all tests.

### Validation

#### Internal consistency reliability

Cronbach’s alpha (α) was used for describing the internal consistency reliability of the CHU9D-DK instrument. The α coefficient was calculated based on the inter-item correlation [33]. The general accepted rule for using Cronbach’s alpha to assess internal consistency is: 0.9 ≤ α excellent; 0.8 ≤ α < 0.9 good; 0.7 ≤ α < 0.8 acceptable; 0.6 ≤ α < 0.7 questionable; 0.5 ≤ α < 0.6 poor; α < 0.5 unacceptable [34]. It was anticipated that the CHU9D-DK would demonstrate acceptable to excellent internal consistency indicating that the items are tapping into the same general construct, i.e. HRQL.

#### Construct validity (known-groups validation)

Known-groups validation was used to examine the extent to which the CHU9D-DK discriminates between groups with known differences. Other studies have demonstrated differences in utility scores by gender, age, clinical conditions and socio-demographic factors [32, 35–37].

##### General health, disability and chronic disease

It was expected a priori that respondents who rated their general health as high with no chronic disease and no disabilities would exhibit higher utility scores according on the CHU9D-DK relative to those who rated themselves in poorer general health overall and with chronic diseases and disabilities [36–38].

##### Life satisfaction

It was expected a priori, that respondents who rated their overall satisfaction with life high would exhibit higher utility scores according to the CHU9D relative to those who rated their overall satisfaction with life low [39].

##### Socio-economic status

Finally, it was expected a priori that students from higher income families, as accessed by the “Money” and “Holiday” variables, would exhibit higher utility scores according to the CHU9D relative to those from lower income backgrounds [19–21, 35, 40].

#### Convergent validity

As both generic instruments, the PedsQL and CHU9D, are designed to measure the same concept, HRQL in young adults/adolescents [32], we hypothesised that there would be conceptual overlap between them and that their related dimensions and overall scores would have a moderate to high correlation. The level of association between the CHU9D and the PedsQL was investigated using Spearman’s correlation coefficient (r). Correlations less than 0.3 were considered weak, 0.3–0.6 moderate, and > 0.6 strong [41]. A dimension level correlation matrix was generated of the CHU9D-DK and the PedsQL instruments combined to assess correlations between like-dimensions, particularly those that were conceptually related.

### Ethics

This study was registered at ClinicalTrials.gov with the identifier: NCT03391999, and can be found at clinicaltrials.gov. The study was also recorded at the Danish Data Protection Agency (study number: 2015-57-0001).

## Results

A total of 228 high school’s students consented to respond and fully completed the questionnaire (participation rate 83.8%). Table 1 presents the characteristics of the study sample, the CHU9D utilities, and the PedsQL scores. The mean age of all 272 students at the high school was 18.45 ± 1.07, and the mean age of the 228 responding/participating students was the same. Among students, there were significantly more boys (62.8%) than girls (37.2%), who fully completed the survey, corresponding to the fact that more boys were attending the high school. The group mean gender differences in HRQL utilities/scores were found to be highly statistically significant (p < 0.0001).

**Table 1:** Mean (SD) CHU9D-utilities and PedsQL-scores by student’s characteristics

	N (%)	CHU9D-utilities - adolescent scoring algorithm applied (best-worst scaling method)	CHU9D-utilities - adult scoring algorithm applied (standard gamble method)	PedsQL-scores
		Mean (SD)	Mean (SD)	Mean (SD)
Whole sample	228	0.70 (0.22)	0.84 (0.11)	82.32 (13.14)
Gender				
Girls	85 (37.2)	0.62 (0.23)	0.80 (0.12)	76.50 (13.45)
Boys	143 (62.8)	0.75 (0.20)	0.86 (0.09)	85.79 (11.68)
*P*-value	* < 0.006^a^	* < 0.001^t^	* < 0.001^t^	* < 0.001^t^
Year of study				
1st year	74 (32.5)	0.73 (0.23)	0.85 (0.12)	84.15 (13.87)
2nd year	83 (36.4)	0.69 (0.22)	0.83 (0.10)	81.05 (12.96)
3rd year	71 (31.1)	0.68 (0.21)	0.83 (0.11)	81.90 (12.52)
*P*-value	0,743^a^	0.394^b^	0.448^b^	0.321^b^
General health				
Excellent	45 (19.7)	0.83 (0.15)	0.91 (0.07)	92.00 (7.40)
Very good	129 (56.6)	0.72 (0.20)	0.84 (0.10)	83.17 (10.70)
Middle	43 (18.9)	0.59 (0.22)	0.79 (0.10)	75.58 (12.84)
Not so good/poor	11 (4.8)	0.42 (0.26)	0.69 (0.14)	59.09 (17.33)
*P*-value	* < 0.001^a^	* < 0.001^b^	* < 0.001^b^	* < 0.001^b^
Disability or chronic disease				
No	202 (88.6)	0.71 (0.21)	0.84 (0.10)	83.50 (12.09)
Yes	26 (12.4)	0.60 (0.26)	0.78 (0.13)	73.20 (17.18)
*P*-value	* < 0.001^a^	*0.012^t^	*0.006^t^	* < 0.001^t^
Satisfaction with life^c^				
Not satisfied (1–3)	19 (8.7)	0.48 (0.22)	0.74 (0.11)	69.91 (17.04)
Medium satisfied (4–7)	65 (30.0)	0.62 (0.23)	0.79 (0.11)	75.08 (13.10)
Very satisfied (8–10)	133 (61.3)	0.77 (0.18)	0.87 (0.09)	86.92 (9.59)
*P*-value	* < 0.001^a^	* < 0.001^b^	* < 0.001^b^	* < 0.001^b^
Money: My family has				
A lot of money	5 (2.2)	0.81 (0.24)	0.89 (0.11)	85.22 (15.46)
A reasonable amount of money	71 (31.1)	0.78 (0.17)	0.87 (0.08)	86.21 (9.93)
Neither a lot or a little amount of money	136 (59.6)	0.66 (0.23)	0.82 (0.11)	80.63 (13.36)
Little or very little money	16 (7.1)	0.64 (0.24)	0.81 (0.12)	78.53 (19.14)
*P*-value	* < 0.001^a^	*0.002^b^	*0.003^b^	*0.017^b^
Holidays last year				
No	45 (19.7)	0.66 (0.21)	0.82 (0.10)	80.12 (13.76)
Yes, one time	93 (40.8)	0.65 (0.23)	0.81 (0.11)	79.74 (13.61)
Yes, several times	90 (39.5)	0.77 (0.19)	0.87 (0.10)	86.10 (11.46)
*P*-value	* < 0.001^a^	* < 0.001^b^	* < 0.001^b^	*0.002^b^

*CHU9D* Child Health Utility 9 Dimension, *PedsQL* Pediatric Quality of Life Inventory™ 4.0 Generic Core Scales, *SD* Standard deviation. ^a^Pearsons Chi^2^-test, ^b^ANOVA, ^c^“don’t know” answers were categorised as missing (*N* = 217), ^t^Two sample t-test. *P < 0.05

For the participating sample, the mean ± SD values of the CHU9D utilities were 0.84 ± 0.11 when the UK adult scoring algorithm was applied and 0.70 ± 0.22, when the AUS adolescent scoring algorithm was applied. For comparison, the mean PedsQL score was 82.32 ± 13.14. CHU9D utilities (both algorithms) and PedsQL scores were not normally distributed (all  p < 0.01).

Table 1 shows, that students who self-reported themselves to have a better general health status, no disability or chronic disease, high life satisfaction, more money and had been on several holidays in the last year, exhibited higher HRQL scores on average for all three instruments relative to other students. Overall, Table 1 also shows that although the direction of the relationships between each instrument and sociodemographic variables was very similar, the mean CHU9D utilities (UK adult scoring algorithm) were on average, higher and exhibited lower SDs than the corresponding PedsQL scores (when PedsQL scores were re-scaled by dividing through by 100). Divergent to this, the corresponding mean utilities (AUS adolescent scoring algorithm) were all lower than the mean PedsQL scores and exhibited higher SDs.

### Internal consistency reliability

Cronbach’s alpha was 0.797 (the standardised Cronbach’s alpha was 0.803), indicating a good level of internal consistency for the CHU9D-DK.

### Known-groups validation

*Hypothesis a. General health, disability, and chronic disease;* there were significant differences in CHU9D utilities (both adult and adolescent scoring algorithms) and PedsQL scores between the levels of self-reported general health, living with or without disability or chronic disease in the expected directions, demonstrating that the two instruments were able to distinguish between groups with self-reported health differences.

*Hypothesis b. Life satisfaction;* Students who indicated that they were very satisfied with their lives had significant higher HRQL utilities (both adult and adolescent scoring algorithms) and PedsQL-scores compared to those who indicated that they were not satisfied with their lives (*p*-values < 0.001).

*Hypothesis c. Socio-economic status;* it was found that students who came from families with higher levels of socio-economic status (as approximated by the “Money” and “Holiday” variables) generally exhibited higher HRQL scores, and these differences were statistically significant for both the PedsQL scores and the CHU9D utilities (p-values < 0.01). One minor exception was in relation to the money variable and the PedsQL instrument where students scored slightly higher on average if they felt they had “a reasonable amount of money” (mean-score 86.21) compared to “a lot of money” (mean-score 85.22). The corresponding CHU9D-scores were however more consistent in this regard.

### Convergent validity

Table 2 summarises the relationship between the dimensions of the CHU9D and the PedsQL regarding Spearman’s correlation coefficients. Overall, as hypothesised, a strong degree of correlation was observed between the two measures (overall r = 0.69;  *p* < 0.001), when the adult scoring algorithm was applied and (overall r = 0.68;  *p* < 0.001), when the adolescent scoring algorithm was applied. At the dimension level, the strongest degree of correlation was found between the ‘Emotional function’ dimension in the PedsQL and the ‘Worried’, ‘Sad’ and ‘Annoyed’ dimensions on the CHU9D (r = 0.47, 0.53 and 0.50, respectively; *p* < 0.001), corresponding to moderate agreement. A moderate degree of correlation was also found between similar dimensions for both instruments, ‘Schoolwork/homework’ in the CHU9D and ‘School functioning’ in the PedsQL (r = 0.38; *p* < 0.001). Finally, a moderate degree of correlation was found between ‘Psychosocial Health’ in the PedsQL and the dimensions ‘Worried’, ‘Sad’ and ‘Annoyed’ in the CHU9D (r = 0.46, 0.43, 0.48, respectively; *p* < 0.001). The weakest degree of correlation was found between the ‘Social function’ in the PedsQL and ‘Pain’, ‘Tired’ and ‘Sleep’ dimensions in the CHU9D (r = 0.21, 0.22 and 0.21, respectively; *p* < 0.001).

**Table 2:** Correlations between CHU9D^1^ dimensions and PedsQL^2^ dimensions

	Worried	Sad	Pain	Tired	Annoyed	Schoolwork Homework	Sleep	Daily routine	Activities	CHU9D utility
Physical Health	0.35	0.38	**0.44**	**0.44**	0.34	0.39	0.38	0.39	**0.48**	0.64 0.62
Psychosocial Health	**0.46**	**0.43**	0.34	0.38	**0.48**	**0.44**	0.37	**0.41**	**0.46**	0.65 0.65
*- Emotional function*	**0.47**	**0.53**	0.38	0.31	**0.50**	**0.41**	0.45	**0.40**	**0.41**	0.68 0.69
*- Social function*	0.28	0.31	*0.21*	*0.22*	**0.38**	0. 31	*0.21*	0.33	0.35	*0.44* 0.43
*- School function*	0.33	0.29	0.26	0.38	0.36	**0.38**	0.26	0.36	**0.40**	0.51 0.50
PedsQL total score	0.45	0.44	0.40	0.43	0.46	0.45	0.40	0.43	0.49	0.69 0.68

*CHU9D* Child Health Utility 9 Dimension, *PedsQL* Pediatric Quality of Life Inventory™ 4.0 Generic Core Scales

The last column shows the CHU9D utility correlations when the adult scoring algorithm (standard gamble-method) was applied, underlined numbers when the adolescent scoring algorithm (best-worst scaling-method) was applied

All Spearman’s correlations are statistically significant (all *p* values < 0.001). The correlations between like dimensions are indicated in boldface. The three least correlated dimensions are indicated in italic

**Fig. 1:**
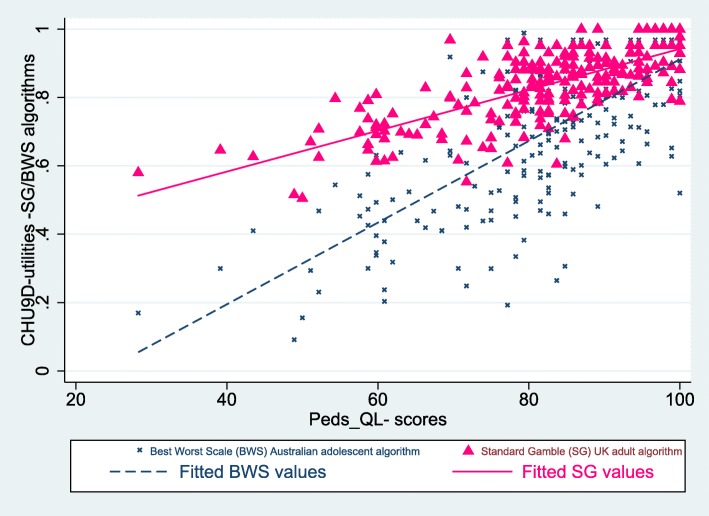
Scatter plot of the CHU9D-DK utilities and the PedsQL-scores, lines showing the corresponding fitted values

Figure 1 presents the scatter plot comparison of the two instruments with the best-fitted line (line fitted by ordinary least squares). For the CHU9D-DK, 17 (7.5%) respondents reported themselves in full health (utility value =1.0, reflecting the best level for all nine dimensions). No participants reported themselves at the worst level of impairment for the CHU9D-DK instrument. For the PedsQL instrument, 17 (7.5%) respondents reported themselves to be in full health (PedsQL = 100), but only five (2.2%) of these were respondents who also reported themselves at full health for the CHU9D-DK instrument.

In general, both scatterplots show moderate agreement between the instruments with the utilities and the scores converging towards the highest end of the scale, where the maximum utility of 1.0 on the CHU9D-DK scale corresponds to the maximum score of 100 on the PedsQL.

**Fig. 2:**
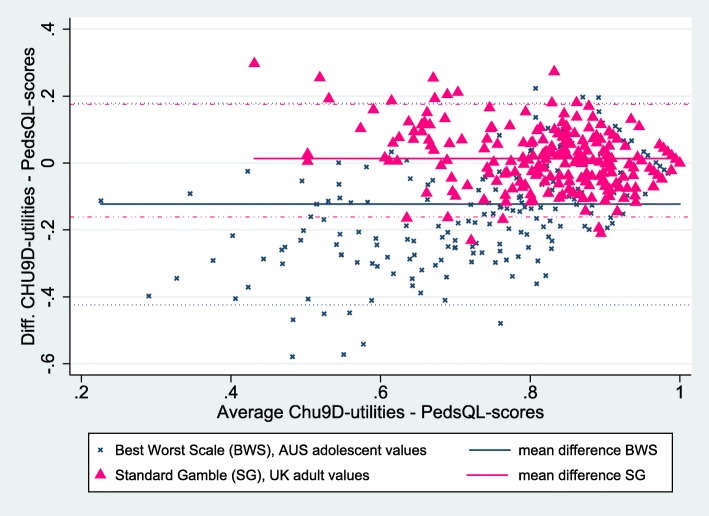
Bland-Altman plot of the CHU9D-utilities and the PedsQL-scores including 95% limits of agreements

Two Bland-Altman scatter plots of the differences between the CHU9D-DK-utilities and the PedsQL-scores (re-scaled by dividing through by 100) are presented in Fig. 2. These two plots also illustrate moderate levels of agreements.

The paired comparison of CHU9D-DK and PedsQL again shows, that the mean CHU9D-DK utilities were on average slightly higher (mean 0.013) than the rescaled PedsQL scores when the adult scoring algorithm was applied) (*p*-value paired t-test =0.025), and lower (mean − 0.123) when the adolescent scoring algorithm was applied (p-value paired t-test < 0.001). As the Bland-Altman plots also show, there were few outliers from the 95% limits of agreement with 13 (5.7%) outliers for the plot based on the adult scoring algorithm for the CHU9D-DK, and ten (4.4%) outliers for the plot based on the adolescent scoring algorithm for the CHU9D-DK.

Table 3 summarises the distribution of mean PedsQL-scores across the dimension levels of the CHU9D-DK. It shows that as expected in a community-based sample of students attending high school, the vast majority of students reported themselves in good health according to the CHU9D-DK.

**Table 3:** Distributions of PedsQL-scores across dimension levels of the CHU9D-DK

CHU9D-DK dimension and levels	Frequency (%)	Mean *PedsQL-scores
*Worried*		
I don’t feel worried today	134 (58.8)	86.87
I feel a little bit worried today	49 (21.5)	80.12
I feel a bit worried today	16 (7.02)	74.12
I feel quite worried today	27 (11.8)	71.46
I feel very worried	2 (0.9)	44.02
*Sad*		
I don’t feel sad today	169 (74.1)	85.84
I feel a little bit sad today	38 (16.7)	73.63
I feel a bit sad	15 (6.6)	74.49
I feel quite sad today	6 (2.6)	57.79
I feel very sad today	0 (0.0)	
*Pain*		
I don’t have any pain today	143 (62.7)	85.92
I have a little bit of pain today	50 (21.9)	79.87
I have a bit of pain today	18 (7.9)	72.28
I have quite a lot of pain today	14 (6.1)	70.73
I have a lot of pain today	3 (1.3)	66.30
*Tired*		
I don’t feel tired today	*29 (12.7)*	91.83
I feel a little bit tired today	*74 (32.5)*	86.02
I feel quite tired today	61 (26.8)	81.00
I feel quite tired today	43 (18.9)	76.09
I feel very tired today	21 (9.2)	72.77
*Annoyed*		
I don’t feel annoyed today	153 (67.1)	86.69
I feel a little bit annoyed today	46 (20.1)	76.87
I feel a bit annoyed today	21 (9.2)	70.60
I feel quite annoyed today	6 (2.6)	63.95
I feel very annoyed today	2 (0.9)	51.63
*School Work/Homework*		
I have no problems with my schoolwork/ homework today	107 (46.9)	87.78
I have a few problems with my schoolwork/ homework today	77 (33.8)	81.23
I have some problems with my schoolwork/ homework today	35 (15.4)	71.65
I have many problems with my schoolwork/ homework today	*7 (3.1)*	*66.15*
I can’t do my schoolwork/homework today	2 (0.9)	76.09
*Sleep*		
Last night, I had no problems sleeping	125 (54.8)	86.4
Last night, I had a few problems sleeping	62 (27.2)	81.9
Last night, I had some problems sleeping	25 (11.0)	73.5
Last night, I had many problems sleeping	14 (6.1)	66.5
Last night, I couldn’t sleep at all	2 (0.9)	62.5
*Daily routine*		
I have no problems with my daily routine today	155 (68.0)	86.3
have a few problems with my daily routine today	48 (21.1)	78.2
I have some problems with my daily routine today	16 (7.0)	71.4
I have many problems with my daily routine today	6 (2.6)	57.2
Can’t do my daily routine today	3 (1.3)	51.8
*Able to join in activities*		
I can join in with any activities today	132 (57.9)	87.8
I can join in with most activities today	52 (22.8)	77.0
I can join in with some activities today	30 (13.2)	73.7
I can join in with a few activities today	9 (4.0)	70.4
I can join in with no activities today	5 (2.2)	66.5

*CHU9D* Child Health Utility 9 Dimension, *PedsQL* Pediatric Quality of Life Inventory™ 4.0 Generic Core Scales

The largest proportion of students reported themselves at the highest dimension level for all dimensions, except for the “Tired” dimension where the highest proportion reported themselves at the second level “I feel a bit tired today”. In general, mean PedsQL-scores corresponded with the CHU9D-DK, with increasing levels of severity on each dimension being associated with lower mean PedsQL-scores. One minor exception was in the School Work/Homework dimension, where seven students reported, “I have many problems with my schoolwork/ homework today” and two students reported, “I can’t do my schoolwork/homework today,” respectively. The seven who reported having many problems on the CHU9D-DK scored lower mean PedsQL (66.15) than the two, who reported, “I can’t do my schoolwork/homework mean PedsQL (76.09). However, only a very small proportion of the total responses exhibited this inconsistency. It is also noticeable that the dimension “Worried” has a more diverse PedsQL range of score, than all other CHU9D-DK dimensions (from 86.87 to 44.02).

## Discussion

To the best of our knowledge, this is the first study in Denmark and Scandinavia to assess the construct validity of the Danish CHU9D-DK instrument. This study compared the measurement properties of the CHU9D-DK with the PedsQL, which is one of the few available generic HRQL instruments linguistically translated into Danish and validated for Danish young people. Both instruments were able to discriminate between students according to their self-reported general health status, living with or without a disability or chronic disease, satisfaction with life and socio-economic status.

Overall, the results of this study indicate that the CHU9D-DK exhibits good construct validity in relation to assessment of the HRQL of high school students in Denmark. Hence, the practical implications are that the CHU9D-DK could potentially be more widely applied with young people in Denmark, especially in the context of health economic evaluations since the CHU9D is a preference-based instrument. However, a limitation in this regard is that no Danish population specific scoring algorithm exists yet. As such, we applied both the existing Australian adolescent scoring algorithm based on the best worst scaling method and the UK adult scoring algorithm based on the standard gamble method. The study shows, as is seen in many other studies, that the utility scores, heavily depend on the measure/algorithm that is used to elicit them [42]. In this study, as has been found elsewhere, systematic differences in utility scores were evident according to the scoring algorithm applied. In general, when the UK adult scoring algorithm was applied, the mean CHU9D-DK utilities were higher and exhibited lower SD than the mean PedsQL scores (the PedsQL rescaled by dividing it by 100). Divergent to this, the mean utilities were lower than the mean PedsQL scores and exhibited higher SD, when the Australian adolescent specific scoring algorithm was applied.

Best practice guidance in the literature indicates that it is preferable to apply country-specific scoring algorithms if available because they better reflect cultural differences and are therefore more likely to represent societal preferences more accurately than scoring algorithms originating from other countries [43–45]. In the future therefore, it will be desirable to develop a Danish valuation set for CHU9D-DK.

The results of this study support the consistency and construct validity of the CHU9D-DK. The level of internal consistency for the CHU9D-DK was good as measured by the Cronbach’s alpha (0.803), and is similar to that found in two other studies, one in Australia [46] and one in China [12], where Chronbach’s alpha was (0.781) and (0.771), respectively. CHU9D-DK utility scores discriminated well in relation to general self-reported health status and life satisfaction. Better general health status and higher life satisfaction were significantly associated with higher utility scores regardless of which scoring algorithm was applied [39]. Further students living with a disability or chronic disease had significantly lower utility scores [36–38]. The same discrimination was seen concerning the two socio-economic questions. The wealthier the family and the more holidays experienced in the preceding year, the higher the utility scores, which is also in line with other studies [19–21, 35, 40].

The findings for this Danish student sample were similar to those reported in another recent Australian study conducted in a similar population of community-based adolescents aged 15–17 years [37]. For example, the CHU9D utility scores were moderately correlated with PedsQL total scores in both studies; although, the overall correlation coefficients found in this study (r = 0.68 (adolescent scoring algorithm) and 0.69 (adult scoring algorithm)) were slightly higher than the Australian study (r = 0.63). The main reason for the moderate correlations between the overall scores generated from the two instruments is that they measure similar concepts [32]. At the dimensional level, however, more discrepancies in correlations are evident between the two instruments. There are various reasons that may explain these discrepancies. Firstly, similar dimensions are not perfectly overlapping in phraseology and descriptions between the two instruments. Secondly, it is severity that is measured in the CHU9D, as compared to frequency in the PedsQL. Thirdly, the recall time is ‘today’ in the CHU9D versus ‘in the past month’ in the PedsQL. Finally, the PedsQL has more items and theoretically covers a broader range of health states than the CHU9D. In contrast whilst the CHU9D has fewer items it includes some unique dimensions not covered by the PedsQL e.g. ‘pain’, which exhibits weak correlations with the PedsQL ‘social’ dimension. Overall, therefore whilst these two instruments are complementary in capturing HRQL, they are also different in terms of how HRQL is described and the time frame applied in HRQL assessment.

### Limitations

There are some limitations to this study. Firstly, the survey was performed in a single high school with a relative small sample size of 228 respondents. This means that all included respondents resided in the same locality and therefore the study sample may not be entirely representative of the Danish high school population in this age group. However, we achieved a high participation rate of 83.8% and this represents a strength of this study.

A second limitation is that the majority of the survey participants were healthy. It is crucial, therefore, to investigate if these findings can be replicated in specific clinical patient samples. Thirdly, the composition of our study sample was such that it fell between two versions of the PedsQL instrument according to age categories, the version for teens (13–18 years) and the version for young adults (18–25 years). We chose not to use two PedsQL versions since the mean age of our sample was very close to 18 years, and opted for the most applicable version for the majority of our sample, the version for young adults.

Whilst the CHU9D is validated for young people aged 7–17 years, it has also been found to perform well in other similar samples of young adults internationally, especially for those in school settings, where the mean age is even higher than in this Danish sample, implying that CHU9D is also valid for application with young adults [22, 23]. The validation results from this study further enrich the evidence base for the applicability of the CHU9D in populations of young adults.

A potential further limitation of our study relates to the measurement of socio-economic status whereby only one of the four items from the Family Affluence Scale, the holiday item was applied. The main reason for not applying the Family Affluence Scale in its entirety was that some of the other items were not so relevant in a Danish setting, e.g., the item “Do you have your own computer?”, since all schoolchildren and young school adults have their own computer in DK. The money item is also a simple way of self-reporting family income and may not represent an accurate reflection of family income in all circumstances. However, this item has been used previously and has been found to perform well in a Danish context [19].

## Conclusions

The findings from this study show that the newly translated and linguistically validated CHU9D-DK demonstrated good psychometric performance overall and shows potential as a valid and reliable instrument for assessing the HRQL of Danish young people. As a preference based instrument, the CHU9D-DK may also be usefully applied in economic evaluations targeted to interventions designed to improve the quality of life of young adults and adolescents in Denmark. Development of a Danish population specific scoring algorithm/s would further facilitate its applicability in this regard.

## Data Availability

The dataset and materials used in this study are available from the corresponding author upon request.

## Appendix

### CHU9D

Thinking about today….

**Table:**

A1. Worried	
O I don’t feel worried today	
O I feel a little bit worried today	
O I feel a bit worried today	
O I feel quite worried today	
O I feel very worried today	
A2. Sad	
O I don’t feel sad today	
O I feel a little bit sad today	
O I feel a bit sad today	
O I feel quite sad today	
O I feel very sad today	
A3. Pain	
O I don’t have any pain today	
O I have a little bit of pain today	
O I have a bit of pain today	
O I have quite a lot of pain today	
O I have a lot of pain today	
A4. Tired	
O I don’t feel tired today	
O I feel a little bit tired today	
O I feel a bit tired today	
O I feel quite tired today	
O I feel very tired today	
A5. Annoyed	
O I don’t feel annoyed today	
O I feel a little bit annoyed today	
O I feel a bit annoyed today	
O I feel quite annoyed today	
O I feel very annoyed today	
A6. Schoolwork/Homework (Such as reading, writing, doing lessons)	
O I have no problems with my schoolwork/homework today	
O I have a few problems with my schoolwork/homework today	
O I have some problems with my schoolwork/homework today	
O I have many problems with my schoolwork/homework today	
O I can’t do my schoolwork/homework today	
A7. Sleep	
O Last night I had no problems sleeping	
O Last night I had a few problems sleeping	
O Last night I had some problems sleeping	
O Last night I had many problems sleeping	
O Last night I couldn’t sleep at all	
A8. Daily routine (Things like eating, having a bath./shower, getting dressed)	
O I have no problems with my daily routine today	
O I have a few problems with my daily routine today	
O I have some problems with my daily routine today	
O I have many problems with my daily routine today	
O I can’t do my daily routine today	
A9. Able to join in activities (Things like playing out with your friends, doing sports, joining in things)	
O I can join in with any activities today	
O I can join in with most activities today	
O I can join in with some activities today	
O I can join in with a few activities today	
O I can join in with no activities today	

## Abbreviations

AUS: Australian
CHU9D: Child Health Utility 9 Dimension
CHU9D-DK: Child Health Utility 9 Dimension Danish version
HRQL: Health Related Quality of Life
PedsQL™: Pediatric Quality of Life Inventory™ 4.0 Generic Core Scales
QALY: Quality Adjusted Life Years
SD: Standard deviation
UK: United Kingdom
